# Value-added green biorefinery co-products from ultrasonically assisted DES-pretreated *Chlorella* biomass

**DOI:** 10.1016/j.ultsonch.2023.106628

**Published:** 2023-09-30

**Authors:** Antira Wichaphian, Nanthakrit Sriket, Sritip Sensupa, Jeeraporn Pekkoh, Wasu Pathom-aree, Yupa Chromkaew, Nakarin Suwannarach, Jaturong Kumla, Benjamas Cheirsilp, Sirasit Srinuanpan

**Affiliations:** a Master of Science Program in Applied Microbiology (International Program), Department of Biology, Faculty of Science, Chiang Mai University, Chiang Mai 50200, Thailand; b Department of Biology, Faculty of Science, Chiang Mai University, Chiang Mai 50200, Thailand; c Biorefinery and Bioprocess Engineering Research Cluster, Chiang Mai University, Chiang Mai 50200, Thailand; d Center of Excellence in Microbial Diversity and Sustainable Utilization, Chiang Mai University, Chiang Mai 50200, Thailand; e Department of Plant and Soil Sciences, Faculty of Agriculture, Chiang Mai University, Chiang Mai 50200, Thailand; f Program of Biotechnology, Center of Excellence in Innovative Biotechnology for Sustainable Utilization of Bioresources, Faculty of Agro-Industry, Prince of Songkla University, Hat Yai, Songkhla 90110, Thailand

**Keywords:** Biorefinery, *Chlorella* Biomass, Biodiesel Feedstock, Hydroponic Biofertilizer, Co-products, Ultrasound-Assisted DES Pretreatment

## Abstract

This study pursued the goal of creating value-added co-products through an environmentally friendly biorefinery approach, employing ultrasonically assisted deep eutectic solvent (DES)-pretreated *Chlorella* biomass. The primary focus was on generating enriched biodiesel feedstock with exceptional fuel properties and developing hydroponic biofertilizer. The results demonstrated the effectiveness of a two-step process involving a 5-minute ultrasound-assisted DES pretreatment followed by ultrasound-assisted solvent extraction, which efficiently extracted lipids from *Chlorella* biomass, yielding biodiesel-quality lipids with good cetane number (59.42) and high heating value (40.11 MJ/kg). Notably, this two-step approach (78.04 mg-lipid/g-microalgal biomass) led to a significant 2.10-fold increase in lipid extraction compared to a one-step process (37.15 mg-lipid/g-microalgal biomass) that combined ultrasound-assisted DES pretreatment and solvent extraction. Importantly, the aqueous extract derived from lipid-extracted microalgal biomass residues (LMBRs) showed promise as a component in hydroponic biofertilizer production, supporting lettuce growth in hydroponic deep water culture system. Consequently, microalgae biorefinery co-products hold tremendous potential in enhancing the profitability and sustainability of interconnected sectors, encompassing renewable energy, agriculture, and the environment.

## Highlights

• A 5-minute ultrasonic treatment, assisted by DES pretreatment, significantly enhanced lipid yields.
• Implementing a two-step lipid extraction process increased the yield by 2.10 times compared to a one-step process.
• The aqueous extract from LMBR can serve as a suitable substitute for fertilizer in hydroponic lettuce cultivation.

## Graphical abstract

**Figure:**
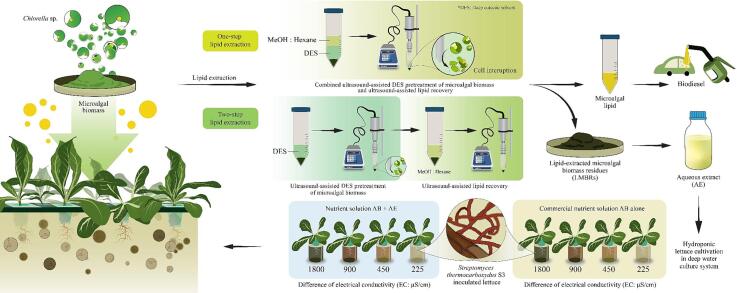

## 1 Introduction

The global scientific community has shown considerable interest in microalgae due to their potential applications in sustainable biotechnological processes, aiming to meet the increasing demand for products derived from natural resources driven by the growing emphasis on healthy habits and fundamental sustainable development [1]. Numerous scientific studies have substantiated the capacity of microalgae to serve as a dependable and renewable source for generating biofuels and valuable bioresources, including polysaccharides, lipids, proteins, enzymes, vitamins, and carotenoids [2], [3]. These investigations have further validated the annual production of 7000 tons of dry microalgae biomass on a global scale [3]. Microalgae biomass represents a valuable natural source of lipids with significant potential as a fuel reserve. Its exploitation is facilitated by certain favorable attributes, including rapid growth, straightforward cultivation needs, and adaptability to challenging conditions [4]. Specific species including *Chlorella* spp., due to their high lipid content ranging from 20 % to 70 %, stand out as attractive candidates for biodiesel production [5]. Nevertheless, the realization of large-scale microalgae biofuel production requires addressing various technical challenges related to harvesting, drying, lipid extraction, and biodiesel manufacturing, as well as overcoming economic hurdles [4]. The improvement of microalgal strains through direct genetic engineering, adaptive laboratory evolution (ALE), and random mutagenesis also deserves attention for achieving high lipid productivity [6]. One of the primary hurdles faced by the biodiesel industry involves the efficient and cost-effective extraction of lipids from microalgae cells. Numerous advanced technologies used to extract lipid composition from microalgae have been employed such as supercritical fluids, ionic liquids, and switchable solvents, as evidenced by the research conducted by Zhou et al. [7] and Khoo et al. [8]. To increase lipid production, it becomes imperative to weaken the thick cell walls of microalgae during the pretreatment stage, as these cell walls consist of intricate polysaccharides and proteins. The primary challenge in sustainable biofuel production lies in developing an effective technique for disrupting microalgae cell walls to facilitate lipid extraction [9]. Various methods are currently employed for cell disruption, falling into two main categories: biochemical (such as alkali/heat or enzymatic treatments) [10] and physical (including bead-beating, osmotic shock, autoclaving, sonication, and microwave methods) [11]. However, these conventional approaches have their limitations, involving high energy consumption and difficulties in scaling due to extreme conditions of pressure and temperature [5], [11]. Furthermore, the conventional lipid extraction methods, whether from wet or dry algae, heavily rely on organic solvents, which pose environmental concerns and are often toxic [5]. Therefore, it becomes imperative to explore alternative approaches that minimize the use of organic solvents and present economic and eco-friendly alternatives for microalgae disintegration as a pretreatment for lipid extraction.

Deep eutectic solvents (DESs), a novel group of eco-friendly solvents, hold the potential to address the issues associated with the costly, environmentally risky, and limiting aspects of conventional solvents. Additionally, DESs offer the advantage of reducing the degradation of target lipids, which often occurs at the elevated temperatures necessitated by organic solvents [12]. Deep eutectic solvents (DESs) are produced through the combination of two or more components, namely, a hydrogen bond acceptor (HBA) and a hydrogen bond donor (HBD). This synthesis leads to a eutectic blend with a melting point lower than that of its individual constituents [5], [12]. In recent times, researchers have explored various types of DESs (choline chloride: oxalic acid, choline chloride: acetic acid, choline chloride: carboxylic acid, choline chloride: urea, and choline chloride: glycerol) as environmentally friendly solvents to weaken the microalga cell wall and facilitate intracellular lipid extraction [5], [12], [13]. The appealing attributes of DESs have prompted some investigations into the combination of DESs with ultrasound to further enhance microalgal lipid extraction, as indicated in the work by Ngatcha et al. [5]. The outcomes of these studies demonstrate a significant improvement in lipid extraction efficiency from microalgal biomass through these pretreatments. However, it is essential to note that the composition and thickness of cell walls vary among different microalgae strains, necessitating distinct fundamental studies for each strain before large-scale implementation. Moreover, in-depth assessments of the physico-chemical properties of the converted biofuel are imperative to ascertain its suitability for practical applications.

Although microalgae hold immense promise as primary contributors to alternative renewable feedstocks, the majority of chemical production processes utilizing microalgal biomass have centered around a singular product, with predominant research efforts directed towards biofuels [14]. Considering a conservative estimation of 25 % extractable oil from microalgal biomass, the production of one metric ton of biodiesel results in three times the quantity of lipid extracted microalgal biomass residues (LMBRs) [15]. The utilization of waste resources, such as waste molasses and municipal wastewater, as a culture medium has the potential to reduce production costs [16], [17]. To ensure the sustainability of biodiesel production, it becomes imperative to find ways to valorize such biomass for various applications, which can also help offset the cost of biodiesel. This added value of LMBRs can be achieved by utilizing them as feed and fertilizer [18], subjecting them to fermentation for biogas and bioethanol production [19], employing them as nutrient sources for organisms [20], utilizing thermo-chemical conversion to produce various fuels and chemicals [21], and using them as biosorbents for the removal of dye and heavy metals from wastewater [15]. Depending on the specific microalgae species, LMBRs are abundant in protein content, resulting in a significant nitrogen presence, alongside other vital plant macro- and micro-nutrients. Due to their low carbon–nitrogen ratio, direct utilization of LMBRs for bio-methane production is impractical. Nonetheless, these biomass residues find potential applications as animal feed, fertilizer, or nutrient sources for various organisms [15]. Previous studies have shed light on the possibility of using microalgae as a source of nutrients and bioactive compounds that can contribute to sustainable plant production, either through direct inoculation [22], utilization of unprocessed dried algae [23], or sonicated biomass [24]. However, thus far, there have been no reported studies that specifically validate the use of LMBRs as a source of plant hydroponic nutrients.

Recently, there has been a growing interest in exploring cascading bio-refineries to fully optimize the inherent value of all components found in microalgae biomass. An approach that shows promise and widespread acceptance for maximizing the utilization of microalgal biomass to create high-value co-products while preserving essential characteristics is the waste-free biorefining strategy [14]. Despite extensive research on microalgal biomass-based biorefineries, none of these studies have investigated the feasibility of utilizing *Chlorella* biomass to simultaneously produce biodiesel with desired fuel properties and hydroponic biofertilizer. Hence, this study was to employ *Chlorella* biomass within a biorefining framework to simultaneously produce biodiesel and biofertilizer. Initially, one- and two-step lipid extraction processes were optimized, incorporating ultrasonic-assisted DESs pretreatment, to maximize the yields of biodiesel feedstock while achieving desired fuel properties. Subsequently, the aqueous extract (AE) obtained from the LMBRs was recovered using ultrasonic-assisted water extraction. The potential of AE as a nutrient substitute for lettuce was evaluated in a laboratory hydroponic deep water culture system, employing various electrical conductivity (EC) levels to assess their impact on lettuce growth, yield, quality, and biochemical properties. To our knowledge, this groundbreaking research project is the first of its kind and possesses the capacity to direct the scientific community toward the valorization of microalgal biomass in tandem with value-added co-products biorefinery as the most effective approach, thereby significantly advancing the Sustainable Development Goals (SDGs) and fostering a more harmonious and prosperous world.

## 2 Materials and methods

### 2.1 Microalgae biomass

Dried microalga *Chlorella* biomass was sourced from the Algal and Cyanobacterial Research Laboratory at Chiang Mai University, Thailand.

### 2.2 Preparation of deep eutectic solvent

The deep eutectic solvent (DES) was prepared following the procedures outlined in Pan et al. [4]. In a 250-mL flask, precise amounts of dried choline chloride (ChCl) and acetic acid (AA) were measured and combined in a 1:3 M ratio. The mixture was continuously stirred in a water bath at 80 °C for 2 h to ensure optimal reaction conditions. This controlled process resulted in the formation of a transparent liquid, indicating the successful synthesis of DES [4]. To prevent moisture absorption, the DES was carefully sealed and stored in a desiccator until it was ready for further experimentation.

### 2.3 One-step of ultrasound-assisted lipid extraction

The extraction of lipids was carried out through a one-step process combining ultrasound-assisted DES pretreatment of microalgal biomass and ultrasound-assisted lipid recovery in a single reaction. The impact of four key factors, including the molar ratio of ChCl:AA, extraction times, microalgal biomass loading, and volume ratio of methanol to hexane, on lipid yields (mg-lipids/g-microalgal biomass) was assessed and optimized using Response Surface Methodology (RSM) with Design Expert version 10.0. The interactive effects of the independent variables were assessed using Central Composite Design (CCD). Each independent variable was varied within the predefined range specified in Table 1. The relationship between the independent variables was established using a second-order polynomial quadratic equation represented as:

(1) $$Y=\beta_{0}+\sum \beta_{i}x_{i}+\sum \beta_{\mathrm{ii}}x_{i}^{2}+\sum \beta_{\mathrm{ij}}x_{i}x_{j}$$

**Table 1:** Central composite design with the experiment response.

Run	Molar ratio of acetic acid to choline chloride	Extraction times (min)	Microalgal biomass loading (g)	Volume ratio of hexane to methanol	Lipid yield (mg-lipid/g-biomass)	
					Experiment value	Predicted value
1	2.00	20.00	0.06	2.00	39.80	33.64
2	3.00	10.00	0.20	1.00	7.41	18.27
3	2.00	20.00	0.40	3.72	7.20	7.54
4	2.00	20.00	0.74	2.00	3.64	9.29
5	0.28	20.00	0.40	2.00	7.46	9.52
6	2.00	20.00	0.40	2.00	9.17	6.72
7	2.00	20.00	0.40	2.00	5.24	6.72
8	2.00	20.00	0.40	2.00	5.22	6.72
9	2.00	2.81	0.40	2.00	12.37	17.87
10	1.00	10.00	0.20	1.00	26.14	23.52
11	1.00	30.00	0.60	3.00	4.14	0
12	1.00	10.00	0.60	1.00	4.98	1.99
13	3.00	30.00	0.60	3.00	3.48	1.33
14	3.00	10.00	0.60	3.00	0.83	5.04
15	1.00	10.00	0.20	3.00	43.91	44.49
16	3.00	30.00	0.60	1.00	9.15	13.71
17	2.00	20.00	0.40	0.28	1.00	0.15
18	3.00	30.00	0.20	1.00	9.38	6.16
19	2.00	37.19	0.40	2.00	4.72	0
20	1.00	30.00	0.20	3.00	5.47	10.02
21	3.00	10.00	0.60	1.00	10.85	1.53
22	3.00	10.00	0.20	3.00	44.46	36.13
23	1.00	30.00	0.20	1.00	3.99	4.93
24	3.72	20.00	0.40	2.00	10.10	7.51
25	1.00	10.00	0.60	3.00	10.15	8.61
26	3.00	30.00	0.20	3.00	0.00	8.13
27	1.00	30.00	0.60	1.00	4.12	7.69

In Equation (1), *Y* represents the experimental response, which corresponds to the lipid yields, while *x_i_* and *x_j_* represent the variables or parameters. The terms *β_0_*, *β_i_*, *β_ij_*, and *β_ii_* represent the offset term, linear effect, first-order interaction effect, and squared effect, respectively. To assess the goodness of fit of the model, the coefficient of determination (*R^2^*) and analysis of variance (ANOVA) were employed. Response surface plots were constructed to determine the optimal condition by holding two independent variables constant while varying the levels of the remaining two variables.

After the completion of the ultrasound process at a frequency of 40 kHz, the addition of hexane facilitated the separation of lipids from the fraction through a phase separation method. Then, the mixture was subjected to vortex and centrifugation at 6000 rpm for 15 min. The upper layer was collected, and the solvent was removed by introducing a nitrogen gas stream. Subsequently, the extracted lipid was dried at a temperature of 60 °C until a constant weight was achieved. The lipid yield was determined as mg-lipid/g-microalgal biomass.

### 2.4 Two-step of ultrasound-assisted lipid extraction

Two stages of ultrasound-assisted lipid extraction were performed, involving ultrasound-assisted DES pretreatment of microalgal biomass and subsequent ultrasound-assisted lipid recovery. In the first stage, the optimal values for the molar ratio of ChCl:AA and microalgal biomass loading, as determined in Section 2.3, were employed to evaluate the efficiency of DES pretreatment. Following ultrasound-assisted DES pretreatment at 40 kHz for 5 and 10 min, the pretreated biomass was separated via centrifugation (at 6000 rpm for 10 min) and combined with the optimal volume ratio of methanol to hexane determined in Section 2.3. The mixture was vortexed and subjected to sonication (at 40 kHz) for 10 min as determined in Section 2.3. After ultrasound-assisted lipid recovery, hexane was added to separate the lipids from the mixture using a phase separation technique described in Section 2.3. The lipid yield was then determined and calculated as mg-lipids/g-microalgal biomass.

### 2.5 Fames characterization and their estimation of fuel properties

The conversion of the extracted lipid into biodiesel, specifically fatty acid methyl esters (FAMEs), was accomplished through the process of acid-catalyzed transesterification. To initiate this transesterification reaction, a precise quantity of the extracted lipid sample (10 mg) was combined with toluene (0.5 mL), methanol (1.5 mL), and concentrated hydrochloric acid (HCl) (50 μL, 35 %). The mixture was thoroughly mixed and subsequently subjected to a controlled heating process at a temperature of 98 °C for a duration of 2 h. Following the completion of the reaction, the mixture was allowed to cool, and a volume of hexane (1 mL) was introduced. Through vigorous shaking, the mixture underwent thorough mixing, resulting in the separation of distinct layers. Specifically, the top layer, known as the FAME layer, containing the fatty acid methyl esters (FAMEs), was meticulously separated and collected for further detailed analysis.

To analyze the composition of the FAMEs, a 7890B Gas Chromatograph equipped with a cross-linked capillary HP-5 column (30 m length, 0.32 mm inner diameter, 0.25 μm film thickness) and a flame ionization detector was employed. The gas chromatograph was operated under specific parameters, including an intake temperature of 230 °C. Initially, the oven temperature was set at 45 °C for a duration of 2 min. Subsequently, a ramp to 100 °C was maintained for 4 min, followed by a ramp to 200 °C maintained for 8 min, a ramp to 250 °C maintained for 6 min, and a detector temperature of 230 °C. By comparing the retention periods of the fatty acids with a set of 37 pure FAME standards, the specific fatty acids present in the sample were identified.

Fatty acid profiling was undertaken to investigate the crucial characteristics of microalgal biodiesel. This comprehensive analysis encompassed the determination of diverse fuel properties, including the saponification value (SV, mg KOH/g), iodine value (IV, g I2/100 g), cetane number (CN), degree of unsaturation (DU, %wt), long-chain saturated factor (LCSF, %wt), cold filter plugging point (CFPP, °C), pour point (PP, °C), high heating value (HHV, MJ/kg), cloud point (CP, °C), kinematic viscosity (υ, ln KV at 40 °C in mm^2^/s), density (ρ, g/cm^3^), oxidative stability (OS; h), allylic position equivalents (APE), and bis-allylic position equivalents (BAPE). The equations described by Pekkoh et al. [14] and Thurakit et al. [25] and were employed for the calculation of these properties.

### 2.6 Recovery of aqueous extract from lipid-extracted microalgal biomass residues (LMBRs)

Aqueous extract was generated from dried lipid-extracted microalgal biomass residues (LMBRs) obtained in Section 2.4. The LMBRs were promptly dissolved in 1000 L of liquid to achieve a final concentration of 2 g/L [26]. Ultrasonic-assisted extraction was employed using an ultrasonic frequency of 35 kHz for a duration of 20 min to recover the aqueous extract from the LMBRs. Subsequently, the mixture was subjected to centrifugation at 6000 rpm for 15 min, and the supernatant was collected. Then, the supernatant was filtered through Whatman filter paper No. 1. Lastly, the aqueous extract was stored at 4 °C to ensure its preservation for future utilization. The basic physical and chemical properties of obtained aqueous extract were as follows: pH, 7.98; electrical conductivity (EC), 193.5 µS/cm; total nitrogen (TN), 52.67 %; extractable nitrate (NO_3_^−^−N), 0.64 mg/L; extractable ammonium (NH_4_^+^−N), mg/L; phosphorus (P), 32.60 mg/L; potassium (K), 9.36 mg/L; sodium (Na), 16.15 mg/L; calcium (Ca), 3.88 mg/L; magnesium (Mg), 16.74 mg/L; iron (Fe), 8.09 mg/L; manganese (Mn), not detectable; copper (Cu), not detectable; zine (Zn), 1.19 mg/L; boron (B), 0.08 mg/L; and sulfur (S), 4.55 mg/L.

### 2.7 Application of LMBRs aqueous extract for lettuce cultivation

Lettuce (*Lactuca sativa* L. var. longifolia) seeds were obtained from the Vegetable Seed Production and Organic Farming Learning Center at Maejo University in Thailand. The seeds were subjected to sterilization by immersing them in 70 % v/v ethanol for 1 min, followed by a 12-minute soak in a 1.2 % v/v NaClO solution. They were then washed three times for 1 min each with sterilized deionized water. After surface sterilization, the lettuce seeds were subjected to inoculation with a spore suspension of *Streptomyces thermocarboxydus* S3 at a concentration of 10^8^ spores/mL. The inoculated seeds were placed on a shaker at room temperature, with a shaking speed of 120 rpm, for 3 h before being sown in the growing tray. The reason for using the inoculation of lettuce seeds with *S. thermocarboxydus* S3 in this research was based on our previous findings. In a previous study, we discovered that the inoculation of rice with *S. thermocarboxydus* S3 helped alleviate the negative impacts of low nutrient and drought stress on rice. Additionally, when mung beans (*Vigna radiata*) were inoculated with this strain, there was a notable improvement in fresh weight, root length, and total length of the plants [27].

The lettuce seeds that had been subjected to inoculation were carefully planted in a growing tray that contained a blend of perlite and vermiculite in a ratio of 3:1. These trays were then positioned within a controlled laboratory environment, characterized by specific conditions. The experimental setup involved an 18-hour photoperiod to ensure sufficient exposure to light, with photosynthetically active radiation reaching an intensity of 2400 lx. The temperature within the laboratory was maintained at a constant 30 °C. To promote healthy growth, the lettuce plants were provided with regular watering on a daily basis. Following a period of seven days from the initiation of seedling growth, when the plants had reached an approximate length of 5 cm and had developed three to four true leaves, they were carefully transplanted into a laboratory hydroponic deep water culture system, and were subsequently grown within a controlled laboratory setting, maintaining the conditions mentioned earlier, for a duration of 28 days. A commercially available hydroponic liquid fertilizer was sourced from Kitsuwan Farm in Notaburi, Thailand. This fertilizer consisted of two solutions: Solution A, containing 115 g/L of Ca(NO_3_)_2_, 2 g/L of 7 % Fe-DTPA, and 4 g/L of 13.2 % Fe-EDTA, and Solution B, consisting of 60 g/L of KNO_3_, 50 g/L of MgSO_4_, 26.5 g/L of KH_2_PO_4_, 5 g/L of Dissolvine® ABC EDTA, and 1 g/L of 13 % Mn-EDTA. These solutions were combined following the manufacturer's instructions. The resultant nutrient solution AB, at full strength, possessed an electrical conductivity (EC) of 1800 µS/cm. The experiments were designed with different EC values and included the following treatments:

- Experiment I: Mixing LMBRs aqueous extract with nutrient solution AB to achieve an EC of 1800 µS/cm.
- Experiment II: Mixing LMBRs aqueous extract with nutrient solution AB to achieve an EC of 900 µS/cm.
- Experiment III: Mixing LMBRs aqueous extract with nutrient solution AB to achieve an EC of 450 µS/cm.
- Experiment IV: Mixing LMBRs aqueous extract with nutrient solution AB to achieve an EC of 225 µS/cm.
- Experiment V: Using nutrient solution AB alone with an EC of 1800 µS/cm.
- Experiment VI: Using nutrient solution AB alone with an EC of 900 µS/cm.
- Experiment VII: Using nutrient solution AB alone with an EC of 450 µS/cm.
- Experiment VIII: Using nutrient solution AB alone with an EC of 225 µS/cm.

At weekly intervals, the EC of all treatments was monitored and adjusted to the designated value for each treatment throughout the experiment by adding the appropriate amount of stock solution. The pH level was also measured every 7 days. After 28 days of cultivation, the lettuces were harvested, and various parameters were assessed, including leaf number, shoot and root length, shoot and root fresh weight, and shoot and root dry weight after drying in an oven at 60 °C until a constant weight was achieved. To analyze chlorophylls and carotenoids, 0.5 g of fresh leaves was socked in 5 mL of 80 % v/v acetone for 24 h. The supernatant was analyzed using spectrophotometry at absorbance wavelengths of 480 nm, 645 nm, and 663 nm. The concentrations of chlorophyll *a*, chlorophyll *b*, total chlorophyll, and carotenoids were calculated using the equations described by Arnon [28].

### 2.8 Statistical analysis

The experiments were conducted with three separate replicates, and the findings are presented as the average value along with the standard deviation. To assess the statistical significance of the results (p-value < 0.05), a one-way analysis of variance (ANOVA) and Duncan's multiple range tests were employed.

## 3 Results and discussion

Microalgal biomass is rich in valuable components, including pigments, lipids, carbohydrates, and proteins, which can be effectively converted into a diverse range of bioproducts through a process called biorefinery. Various industries, such as food, energy, nutraceuticals, and agriculture, can greatly benefit from the implementation of this approach. Given that the economic viability of microalgal bioproducts cannot solely rely on the recovery of a single product, it is necessary to sequentially separate the biomass to maximize its potential. Hence, this study was conducted within the framework of a biorefinery approach, which aimed to minimize waste generation. The research focused on extracting two valuable co-products, namely biodiesel feedstock and hydroponic liquid fertilizer, from microalgal *Chlorella* spp. biomass using an integrated biorefining process. The biorefining process consisted of two main steps: (I) extracting lipids to produce high-quality biodiesel with excellent fuel properties, and (II) extracting an aqueous extract from the lipid-extracted microalgal biomass residues (LMBRs) to create hydroponic liquid fertilizer with the potential to promote plant growth.

### 3.1 Value-Added green biorefinery product I: Enriched biodiesel feedstock with exceptional fuel properties

In this study, the primary value-added biorefinery product is microalgal biodiesel characterized by its exceptional fuel properties. As widely acknowledged, one of the major hurdles in the biodiesel industry is the efficient, environmentally friendly, rapid, and cost-effective extraction of internal lipids from microalgae cells. The complex and rigid cell wall structures make it challenging to liberate the lipids from the cells. In this part we employed a one-step process combining ultrasound-assisted DES pretreatment of microalgal biomass and ultrasound-assisted lipid recovery in a single reaction. The performance of this extraction method was compared to a two-stage process that involved ultrasound-assisted DES pretreatment of microalgal biomass and subsequent ultrasound-assisted lipid recovery.

#### 3.1.1 One-step of ultrasound-assisted lipid extraction

Response surface methodology (RSM) was employed in this study to optimize the extraction conditions for lipids from *Chlorella* biomass. The investigation focused on key factors, namely the molar ratio of acetic acid and choline chloride (A), extraction times (B) measured in minutes, microalgal biomass loading (C) in grams, and the volume ratio of hexane to methanol (D). These variables were systematically varied within specified ranges, as outlined in Table 1. The experimental outcomes were centered on lipid yield (Y), quantified as milligrams of lipids per gram of microalgal biomass. A four-factor central composite design (CCD) experimental design (Table 1) was employed. The response equations utilized second-order polynomials to establish predictive models, enabling the determination of empirical relationships between the independent variables and lipid yield, as exemplified below.

Y = 51.72 – 7.09A – 1.51B – 172.02C + 22.73D + 0.16AB + 5.99AC – 0.78AD + 3.04BC – 0.40BD – 17.95CD + 0.61A^2^ + 5.28 × 10^–3^B^2^ + 124.73C^2^ – 0.98D^2^ (2).

As indicated by the Equation (2), the negative sign preceding each coefficient term indicates an antagonistic effect, whereas a positive sign suggests a synergistic effect. The models developed in this study can be utilized to predict the values of the responses based on various combinations of independent variables within the experimental range. The significance of these models was assessed using analysis of variance (ANOVA) to determine their statistical validity. Typically, a model is considered significant if it satisfies the following criteria: a significant *F*-value greater than 0.1, an R^2^ value approaching 1, a *P*-value less than 5 %, and an insignificant lack of fit [20]. According to the ANOVA analysis (Table 2), the response model was found to be statistically significant with a *p*-value less than 0.05. Additionally, the lack of fit's *p*-value was greater than 0.05, indicating that the lack of fit was not considered significant. The lack of fit test compares the residual error with the pure error originating from center points. The lack of fit is calculated from the difference between residual error and pure error. The lack of fit in the model, as indicated by the *F*-value of 12.11 (Table 2), was determined to be not statistically significant compared to the pure error. This confirms that the model is well-fitted. Furthermore, the ANOVA results indicated that the model exhibited a satisfactory relationship between the response and independent variables, as evidenced by the high *F*-value and low p-value (Table 2). Previous studies have emphasized the importance of having a large *F*-value and a small p-value to confirm the significance of the coefficient terms [29]. In this study, the model yielded an *F*-value of 4.61 (Table 2), suggesting that a significant portion of the variations in the response can be explained by the regression equation. Moreover, the R^2^ value achieved in this study for the model was 0.8432 (Table 2), indicating that the quadratic model reliably predicted approximately 84.32 % of the response. This was supported by the strong correlation and agreement observed between the experimental values and the values calculated from the quadratic model [30]. Additionally, the adequacy of precision was evaluated by the signal-to-noise ratio, which should exceed 4.0 to indicate a sufficiently discriminative model [29]. The obtained value of adequate precision for the model was 42.22 (Table 2), suggesting that the developed model can effectively navigate the designated parameter space.

**Table 2:** ANOVA for response surface quadratic model of response variance.

Source	Sum of Squares	df	Mean Square	*F*-value	*p*-value	
Model	3427.43	14	244.82	4.61	0.0058	Significant
A	7.49	1	7.49	0.14	0.7138	Not significant
B	680.94	1	680.94	12.82	0.0038	Significant
C	1099.33	1	1099.33	20.69	0.0007	Significant
D	101.2	1	101.2	1.9	0.1927	Not significant
AB	41.98	1	41.98	0.79	0.3915	Not significant
AC	22.97	1	22.97	0.43	0.5232	Not significant
AD	9.7	1	9.7	0.18	0.6767	Not significant
BC	590.1	1	590.1	11.11	0.0060	Significant
BD	252.27	1	252.27	4.75	0.0500	Significant
CD	206.1	1	206.1	3.88	0.0724	Not significant
A^2^	5.49	1	5.49	0.1	0.7535	Not significant
B^2^	4.15	1	4.15	0.078	0.7845	Not significant
C^2^	370.82	1	370.82	6.98	0.0215	Significant
D^2^	14.18	1	14.18	0.27	0.6148	Not significant
Residual	637.57	12	53.13			
Lack of Fit	627.22	10	62.72	12.11	0.0786	Not significant
Pure Error	10.35	2	5.18			
Cor Total	4065	26				
R^2^	0.8432					
Adequate Precision	8.48					

The results of ANOVA (Table 2) also indicated that the linear terms of extraction times (B) and microalgal biomass loading (C) had a significant impact on the lipid yield, with *p*-values below 0.05. This suggests that achieving an optimal lipid yield requires careful consideration of the appropriate extraction time and microalgal biomass loading. Moreover, the interactions between the independent variables of extraction times and microalgal biomass loading (BC) and extraction times and volume ratio of hexane to methanol (BD), as well as the quadratic term of microalgal biomass loading (C^2^), were also found to be statistically significant at a level below 5 %. These favorable interactions further contribute to maximizing the lipid yield. According to the findings from the experimental results (Table 2), the lipid yield varied between 0 and 44.46 mg-lipid/g-microalgal biomass. Among the runs, Run 22 exhibited the highest lipid yield of 44.46 mg-lipid/g-microalgal biomass. This run involved a molar ratio of acetic acid to choline chloride of 3:1, an extraction time of 10 min, a microalgal biomass loading of 0.2 g, and a volume ratio of hexane to methanol of 3:1 g/L.

**Fig. 1:**
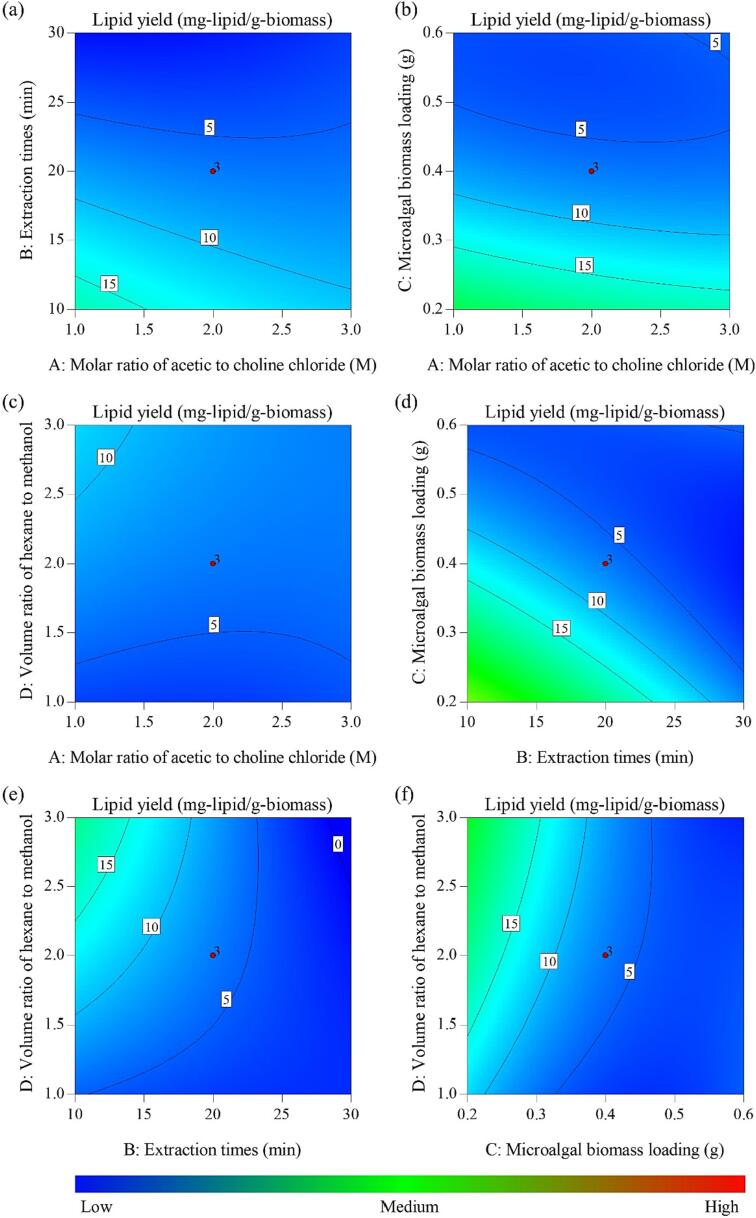
2D contour plots illustrating the impact of the molar ratio of acetic acid to choline chloride, extraction times, microalgal biomass loading, and volume ratio of hexane to methanol on the lipid yield. In these plots, two variables were maintained at their center point while the other two variables were varied within the experimental range.

Fig. 1 displays contour plots generated from the Equation (2), where two variables are held constant at its center point while the other two variables are varied within their respective ranges. These plots were created to examine the interactions between the independent variables and determine the optimal levels for achieving the desired response in each variable. The results indicate that a higher lipid yield can be attained by using a higher molar ratio of acetic acid to choline chloride, a shorter duration for ultrasonic-assisted lipid extraction, a greater microalgal biomass loading, and a higher volume ratio of hexane to methanol. These findings suggest that these conditions are favorable for efficient lipid extraction. Several studies in the literature support the notion that optimizing certain parameters can lead to a higher lipid yield during ultrasonic-assisted lipid extraction. Firstly, the molar ratio of acetic acid to choline chloride has been found to have a significant impact on lipid extraction efficiency. Research by Pan et al. [4] demonstrated that increasing the molar ratio of acetic acid to choline chloride resulted in higher lipid yields from microalgal biomass. This can be attributed to the ability of the DES to solubilize lipids and facilitate their extraction from the biomass. The DES composition plays a crucial role in breaking down the cell walls and promoting lipid release, ultimately leading to an increased lipid yield. Matchim Kamdem et al. [12] discovered that the presence of acidic conditions enhances the breakdown of microalgae cell walls and leads to improved lipid extraction. Consequently, the interaction between anions, hydroxyl groups, carboxyl groups, or acylamino groups in the DES and hydroxyl in the cell membrane may have the potential to modify the hydrogen bonds of cellulose and hemicellulose in the cell walls of *Chlorella* sp. microalgae [31].

Secondly, the duration of ultrasonic-assisted lipid extraction has been identified as an influential parameter. The application of ultrasound waves aids in disrupting cell structures and enhancing mass transfer, enabling efficient extraction of lipids [32]. However, excessive extraction times may lead to degradation or loss of lipids, thereby compromising the overall yield [33]. Studies by Pan et al. [4] showed that shorter extraction times resulted in higher lipid yields. Furthermore, the microalgal biomass loading, or the amount of biomass used for extraction, has been reported to impact lipid extraction efficiency. Studies conducted by Ngatcha et al. [5] have shown that augmenting the amount of microalgal *Chlorella* biomass used in the process resulted in elevated lipid yields. However, it was observed that further increases in biomass loading did not lead to additional improvements in the overall lipid yields. This can be attributed to the higher availability of lipids for extraction at greater biomass concentrations. However, there may be an upper limit beyond which the excess biomass may hinder efficient mass transfer [5]. Lastly, the volume ratio of hexane to methanol has been recognized as a significant factor affecting lipid yield. Studies by Pan et al. [4] reported that a higher volume ratio of hexane to methanol resulted in increased lipid yields. Hexane is a non-polar solvent that exhibits excellent affinity for lipids, while methanol aids in breaking the interactions between lipids and other cellular components. Therefore, a higher volume ratio of hexane to methanol enhances the solubilization and extraction of lipids, leading to improved lipid yield.

Overall, the degree of importance for the four key parameters affecting the extraction performance can be summarized as follows: microalgal biomass loading > extraction times > volume ratio of hexane to methanol > molar ratio of acetic acid and choline chloride. Regarding the interaction effects, the relative importance is as follows: extraction times vs. microalgal biomass loading > extraction times vs. volume ratio of hexane to methanol > microalgal biomass loading vs. volume ratio of hexane to methanol > molar ratio of acetic acid and choline chloride vs. extraction times > molar ratio of acetic acid and choline chloride vs. microalgal biomass loading > molar ratio of acetic acid and choline chloride vs. volume ratio of hexane to methanol. The ideal conditions for achieving the highest lipid yield were determined by calculating the partial derivatives of Equation (2) and setting them to zero. These conditions included a molar ratio of acetic acid to choline chloride of 3:1, an extraction time of 10 min, a microalgal biomass loading of 0.2 g, and a volume ratio of hexane to methanol of 3:1. The maximum estimated lipid yield was found to be 36.13 mg-lipid/g-biomass. To verify the results, three replicate experiments were conducted under optimal conditions, which yielded a biomass productivity of 37.15 ± 1.32 mg-lipid/g-biomass. The experimental values closely matched the predicted values and showed no significant difference (P < 0.05).

The combination of ultrasound-assisted DES pretreatment and ultrasound-assisted solvent extraction in a biphasic extraction system enhances the efficiency of lipid extraction from microalgal biomass. The ultrasound waves aid in disrupting the cell walls during the pretreatment step, improving access to intracellular lipids [32]. The biphasic system allows for selective partitioning of lipids into the organic solvent phase, aided by the ultrasound waves that enhance mixing and mass transfer [34]. However, although the application of RSM to optimize the conditions for lipid extraction yielded positive results, the obtained lipid yield fell short compared to the findings reported by Pan et al. [4] and Ngatcha et al. [5]. They recorded a maximum lipid yield of up to 300 mg-lipid/g-biomass after a one-step extraction using a combination of ultrasound-assisted DES pretreatment and ultrasound-assisted solvent extraction in a biphasic extraction system. This disparity in outcomes could be attributed to the potential interference of components within the extraction system. Biphasic extraction, which involves the incorporation of two immiscible phases, typically DES phase and a solvent phase, introduces the complexity of multiple components and phases that can influence the solubility and extraction efficiency of lipids. Interactions between the DES and solvent phases, along with the presence of impurities or contaminants, have the potential to impede the extraction process, subsequently leading to a reduction in lipid yield. Another plausible explanation for the lower lipid yield is the selectivity limitations associated with biphasic extraction techniques. These methods may not exhibit high selectivity towards specific lipid types, resulting in the extraction of a broad range of lipids, including polar and non-polar lipids. Consequently, a mixture of lipids with diverse properties is obtained, leading to a lower overall lipid yield, and potentially impacting the purity and quality of the extracted lipids. However, variations in lipid yields can be attributed to species-specific differences and the complex interplay between lipid extraction techniques and pretreatment methodologies [5].

#### 3.1.2 Enhancing the performance of ultrasound-assisted lipid extraction using two-step process

To maximize lipid yields, a comprehensive investigation employing a two-step process of ultrasound-assisted lipid extraction was carried out. This process involved the initial step of ultrasound-assisted DES pretreatment of microalgal biomass, followed by the subsequent step of ultrasound-assisted solvent extraction. The influence of varying pretreatment times, specifically 5 and 10 min, on lipid yields was carefully evaluated. Subsequently, the obtained lipid yields from the two-step extraction process were meticulously compared with those attained through the one-step extraction approach. The comprehensive results and corresponding data can be found in Table 3. The findings of the study revealed that pretreatment durations of 5 and 10 min led to notable improvements in lipid yields, with recorded values of 78.04 and 75.04 mg-lipid/g-microalgal biomass, respectively. These results significantly surpassed the lipid yields obtained through the one-step extraction process, which yielded only 37.15 mg-lipid/g-microalgal biomass. This observed enhancement in lipid yields can be attributed to the effectiveness of pretreatment in facilitating enhanced contact between the intracellular lipid content and the solvent, consequently improving the overall lipid extraction efficiency [35]. However, the lipid yields were not enhanced by a longer ultrasonic-assisted DES pretreatment time (10 min) compared to a 5-minute pretreatment. These findings align with the results reported by Ngatcha et al. [5], who also observed that longer ultrasonic times resulted in lower lipid yields. Ultrasonication operates by exploiting the phenomenon of cavitation, which encompasses the formation of minute bubbles within the fluid. These bubbles undergo expansion during the rarefaction phase and compression during the compression phase. The ensuing collapse of these bubbles produces vigorous shock waves accompanied by the simultaneous release of heat, pressure, and shear stress, culminating in the disruption of cells. Typically, this procedure leverages water as a medium to facilitate the generation of microbubbles. However, the utilization of DES introduces a challenge due to its elevated viscosity, which impedes the formation and expansion of microbubbles with increasing pretreatment time. This heightened viscosity is necessary to enable the emulsification of biomass and DES [5], [36]. Consequently, the biomass is not adequately pretreated, resulting in a decline in lipid yield when subjected to prolonged ultrasonic processing with DES without the addition of water. Despite the absence of improvement in lipid yield with increased ultrasonic time, the two-step extraction process yielded approximately twice as much lipid as the one-step extraction process. Therefore, it can be concluded that the most effective approach for extracting lipids from *Chlorella* microalgal biomass involves a two-stage process of ultrasound-assisted lipid extraction. This process includes a 5-minute ultrasound-assisted DES pretreatment of the microalgal biomass, followed by ultrasound-assisted solvent extraction.

**Table 3:** Lipid yields and fatty acid composition of produced biodiesel.

Parameters	One-step	Two-step	
Pretreatment time		5 min	10 min
Lipid yield (mg-lipid/g-microalgal biomass)	37.15 ± 1.32	78.04 ± 1.77	75.04 ± 2.65
Fatty acids	Relative content (%)		
Caproic acid (C6:0)	0.10 ± 0.01	0.05 ± 0.00	0.05 ± 0.01
Caprylic acid (C8:0)	0.04 ± 0.00	ND	ND
Capric acid (C10:0)	0.02 ± 0.01	0.01 ± 0.00	0.01 ± 0.00
Lauric acid (C12:0)	0.09 ± 0.01	0.09 ± 0.03	0.07 ± 0.01
Myristic acid (C14:0)	0.50 ± 0.01	0.47 ± 0.03	0.44 ± 0.01
Myristoleic acid (C14:1)	0.13 ± 0.01	0.16 ± 0.00	0.13 ± 0.01
Pentadecanoic acid (C15:0)	0.93 ± 0.02	1.04 ± 0.00	1.13 ± 0.06
Palmitic acid (C16:0)	29.88 ± 0.29	29.01 ± 0.13	28.58 ± 0.29
Palmitoleic acid (C16:1)	3.47 ± 0.06	4.06 ± 0.02	4.09 ± 0.12
Heptadecanoic acid (C17:0)	0.43 ± 0.04	0.54 ± 0.02	0.51 ± 0.00
*cis*-10-Heptadecanoic acid (C17:1)	1.09 ± 0.00	1.17 ± 0.00	1.19 ± 0.00
Stearic acid (C18:0)	3.11 ± 0.02	2.74 ± 0.02	2.30 ± 0.02
Elaidic acid (C18:1n9t)	45.07 ± 0.49	47.29 ± 0.65	47.67 ± 1.35
Oleic acid (C18:1n9c)	14.10 ± 0.07	12.15 ± 0.91	12.51 ± 0.87
γ-Linolenic acid (GLA) (C18:3n6)	0.08 ± 0.02	ND	ND
Arachidic acid (C20:0)	0.21 ± 0.00	0.22 ± 0.00	0.22 ± 0.00
*cis*-11-Eicosenoic acid (C20:1n9)	0.11 ± 0.01	0.13 ± 0.00	0.13 ± 0.00
Eicosadienoic acid (C20:2n6)	0.08 ± 0.01	0.09 ± 0.00	0.10 ± 0.00
*cis*-8,11,14-Eicosatrienoic (C20:3n6)	0.07 ± 0.01	0.09 ± 0.00	0.09 ± 0.00
Arachidonic acid (C20:4n6)	0.07 ± 0.09	0.17 ± 0.00	0.16 ± 0.00
Henicosanoic acid (C21:0)	ND	0.03 ± 0.00	0.02 ± 0.00
Behenic acid (C22:0)	0.06 ± 0.00	0.07 ± 0.00	0.10 ± 0.02
Erucic acid (C22:1n9)	ND	0.01 ± 0.00	0.01 ± 0.00
13,16-Docosadienoic acid (C22:2n6)	ND	0.01 ± 0.00	ND
*cis*-4,7,10,13,16,19-Docosahexaenoic (C22:6n3)	0.03 ± 0.05	0.05 ± 0.00	0.07 ± 0.02
Tricosanoic acid (C23:0)	0.01 ± 0.01	0.03 ± 0.00	0.04 ± 0.01
Lignoceric acid (C24:0)	0.33 ± 0.02	0.33 ± 0.01	0.38 ± 0.01
C16-C18	97.23 ± 0.14	96.96 ± 0.07	96.84 ± 0.09
Saturated fatty acids (SFAs)	35.17 ± 0.27	34.63 ± 0.23	33.85 ± 0.37
Unsaturated fatty acids (UFAs)	64.29 ± 0.27	65.37 ± 0.23	66.15 ± 0.37
Monounsaturated fatty acids (MUFAs)	63.96 ± 0.36	64.99 ± 0.24	65.75 ± 0.34
Polyunsaturated fatty acids (PUFAs)	0.33 ± 0.09	0.38 ± 0.01	0.04 ± 0.02

ND is not detectable.

Table 4 provides a comparative analysis of lipid extraction yields achieved through both one-step and two-step processes, drawing insights from prior research. Our results exhibit a degree of alignment with previously documented lipid yields from various microalgal biomass [5], [12], [13], [37], [38]; however, they manifest variations in magnitude. This variability can likely be attributed to the choice of distinct microalgal biomass specimens, their inherent capacity for lipid accumulation, and the specificities of the employed extraction methodologies. While the optimized methodology employed in this study has demonstrated the efficiency of microalgal lipid extraction, it is noteworthy that several critical parameters, including the selection of solvents, ultrasonic duration, and ultrasonic frequency, necessitate further refinement in forthcoming investigations. This optimization process is vital for the purpose of maximizing lipid yields during ultrasound-assisted solvent extraction.

**Table 4:** Comparison with previous studies on lipid yields by one-step and two-step extraction processes.

Process	Species	Pretreatment and extraction method	DES (molar ratio)	Lipid yield (mg-lipid/g-microalgal biomass)	Reference
One-step	*Dunaliella salina*	DES assisted solvent extraction	Choline chloride-urea (1:2)	338.8	[13]
	*Nannochloropsis* sp.	Three-phase partitioning	Tetramethylguanidine-methol (3:1)	172	[37]
	*Chlorella* sp.	Ultrasound-assisted DES pretreatment	Choline chloride-Acetic acid (1:3)	37.15	This study
Two-step	*Chlorella pyrenoidosa*	DES pretreatment and solvent extraction Pretreatment 5 min Pretreatment 10 min	Choline chloride-Acetic acid (1:3)	59 63.5	[5]
	*Chlorella pyrenoidosa*	Ultrasound-assisted DES pretreatment and solvent extraction Pretreatment 5 min Pretreatment 10 min	Choline chloride-Acetic acid (1:3)	96 64	[5]
	*Chlorella pyrenoidosa*	DES pretreatment and sonication extraction	Choline chloride-malonic acid (1:1)	169.7	[12]
	*Dunaliella salina*	DES pretreatment and solvent extraction	Choline chloride-urea (1:2)	749.9	[13]
	*Chlorella* sp.	Ultrasound-assisted DES pretreatment and Bligh and Dyer’s extraction	Choline chloride-urea (1:2)	510	[38]
	*Chlorella* sp.	Ultrasound-assisted DES pretreatment and ultrasonication extraction 40 kHz, 5 min 40 kHz, 10 min	Choline chloride-Acetic acid (1:3)	78.04 75.04	This study

#### 3.1.3 FAME characterization and estimation of fuel properties

Upon analyzing the FAME profiles of biodiesel derived from microalgal lipid (as shown in Table 3), it can be observed that both the one-step and two-step approaches have minimal impact on the FAME characteristics. This suggests that the optimized two-step method, involving a 5-minute DES pretreatment of microalgal biomass, is suitable for efficient lipid extraction prior to biodiesel production. The FAME mixture consists of a total of 27 fatty acids, with the majority being C16 to C18 methyl esters. The predominant fatty acids identified in the overall FAME composition were palmitic acid (C16:0), palmitoleic acid (C16:1), stearic acid (C18:0), elaidic acid (C18:1n9t), and oleic acid (C18:1n9c), which collectively represented over 95 % of the total FAME content. This composition met the specifications for biodiesel production, and the inclusion of short-chain fatty acids (alkyl chains ranging from C12 to C18) was essential for biodiesel properties [14]. In accordance with the European biodiesel standard, the levels of fatty acids with more than four double bonds should not exceed 1 %, and linoleic acid (C18:3) should be less than 12 % [20]. The findings of this study indicate compliance with these criteria, as the identified fatty acids primarily consisted of a minimal quantity of polyunsaturated fatty acids (PUFAs) with four or more double bonds, accounting for less than 0.4 %. Additionally, the presence of C18:3 fatty acid was observed at only 0.08 % in the one-step process (Table 3), while it was not detectable in the two-step process.

Table 3 also categorized the FAME constituents into saturated fatty acids (SFAs), monounsaturated fatty acids (MUFAs), and polyunsaturated fatty acids (PUFAs). The results revealed that MUFAs accounted for the highest proportion (63.96–65.75 %), followed by SFAs (33.85–35.17 %), and PUFAs (0.04–0.38 %), respectively. The inclusion of monounsaturated fatty acids (MUFA) and polyunsaturated fatty acids (PUFA) in biodiesel offers the advantage of having a lower melting point, enabling better fuel flow in cold temperatures compared to saturated fatty acids (SFA) [39]. But the presence of these FAME species requires the use of an oxidative stabilizer for safe application [29]. The susceptibility of PUFAs and MUFAs to autoxidation contributes to the degradation of biodiesel and potential complications in the fuel system [29], [40]. Nevertheless, the proportion of SFAs, MUFAs, and PUFAs obtained in this study closely resembled those found in other biodiesel feedstocks, including widely utilized industrial feedstocks like palm oil and Jatropha [41]. More interestingly, over 99 % of the microalgal lipids extracted in this study comprised SFAs and MUFAs. This suggests that utilizing these extracted lipids from *Chlorella* as a feedstock for biodiesel production would result in an eco-friendly biodiesel with several advantages, including a higher cetane number, reduced NOx emissions, shorter ignition delay time, and improved oxidative stability. Pekkoh et al. [20] reported comparable results, highlighting the importance of higher concentrations of SFAs and MUFAs in enhancing the fuel properties of biodiesel. These findings indicate that the microalgal lipids extracted in this study hold promise as a viable feedstock for biodiesel production.

On the other note, the establishment of biofuel standards is crucial for ensuring the market acceptance of biodiesel, as there can be significant variations in quality, largely influenced by the compositions of FAMEs [14]. Hence, the fuel properties of biodiesel obtained from microalgal lipids through both one-step and two-step extraction methods are assessed and compared to industry standards (EN 14,214 and ASTM D6751), as presented in Table 5. These include saponification value (SV), iodine value (IV), cetane number (CN), degree of unsaturation (DU), long-chain saturated factor (LCSF), cold filter plugging point (CFPP), cloud point (CP), pour point (PP) high heating value (HHV), kinematic viscosity (υ), density (ρ), allylic position equivalents (APE), bis-allylic position equivalents (BAPE), oxidative stability (OS). Results demonstrated that the fuel properties of biodiesel derived from microalgal lipids remained unchanged regardless of whether the one-step or two-step extraction method was employed. The SV, IV, CN, DU, LCSF, CFPP, CP, PP, HHV, υ, ρ, APE, BAPE, and OS exhibited values within the range of 205–206 mg-KOH/g-oil, 58–61 g I_2_/100 g-oil, 59–60, 64–67 %wt, 5–6 %wt, −0.4–0.8 °C, 10–11 °C, 4–5 °C, 40–41 MJ/kg, 5–6 ln KV at 40 °C in mm^2^/s, 0.8–0.9 g/cm^3^, 118–121, 0.0–0.2, and > 17.7 h, respectively. These values indicate excellent resistance to oxidation [42], as well as high calorific value [14] and favorable ignition characteristics with a shorter ignition time [25]. Importantly, the fuel properties of microalgal lipids conform to the well-established international standards ASTM D6751 and EN 14214 (Table 5), thereby substantiating their suitability as a promising feedstock for biodiesel production.

**Table 5:** Estimated fuel properties of biodiesel derived from microalgal lipids using one-step and two-step extraction processes.

Parameters	One-step	Two-step		International standards	
		5 min	10 min	EN 14,214	ASTM D6751
SV	205.67 ± 0.10	205.33 ± 0.07	205.16 ± 0.11	NS	NS
IV	58.64 ± 0.13	59.83 ± 0.18	60.59 ± 0.38	≤120	NS
CN	59.64 ± 0.04	59.42 ± 0.03	59.27 ± 0.07	≥51	≥47
DU	64.62 ± 0.18	65.77 ± 0.22	66.57 ± 0.39	NS	NS
LCSF	5.50 ± 0.01	5.25 ± 0.05	5.14 ± 0.02	NS	NS
CFPP	0.80 ± 0.04	0.02 ± 0.15	−0.34 ± 0.08	≤5	NS
CP	10.72 ± 0.15	10.27 ± 0.07	10.04 ± 0.15	>4	NS
PP	4.82 ± 0.16	4.33 ± 0.07	4.08 ± 0.16	3 to 15	NS
HHV	40.12 ± 0.01	40.11 ± 0.00	40.11 ± 0.00	NS	NS
υ	5.51 ± 0.93	5.31 ± 0.19	5.10 ± 0.00	3.5 to 5.0	1.9 to 6
ρ	0.85 ± 0.00	0.85 ± 0.00	0.85 ± 0.00	0.8 to 0.9	NS
APE	118.49 ± 0.88	118.88 ± 0.51	120.35 ± 0.95	NS	NS
BAPE	0.16 ± 0.04	0.00 ± 0.00	0.00 ± 0.00	NS	NS
OS	>17.7	>17.7	>17.7	≥8	≥3

SV is the saponification value (mg KOH/g); IV is the iodine value (g I_2_/100 g), CN is the cetane number; DU is the degree of unsaturation (%wt); LCSF is the long-chain saturated factor (%wt); CFPP is the cold filter plugging point (°C); CP is the cloud point (°C); PP is the pour point (°C); HHV is the high heating value (MJ/kg); υ is the kinematic viscosity (ln KV at 40 °C in mm^2^/s); ρ is the density (g/cm^3^); APE is the allylic position equivalents; BAPE is the bis-allylic position equivalents; OS is oxidative stability (h); EN is the European standard of biodiesel; ASTM is the American Society for Testing and Materials (American standard of biodiesel); NS is not specified.

Based on the aforementioned results, while the lipid extraction process does not have a significant impact on the quality of biodiesel, it is worth noting that the utilization of a two-step process, incorporating a 5-minute ultrasound-assisted DES pretreatment of microalgal biomass followed by ultrasound-assisted solvent extraction, demonstrates superior performance in extracting lipids from microalgal *Chlorella* biomass. This suggests that the lipid obtained through this process is an ideal feedstock for the production of high-quality biodiesel. In addition, the biodiesel produced in this study can be utilized as a component in biodiesel blends. Biodiesel blends are recognized as an alternative approach to reduce the reliance on fossil fuels for biodiesel production. These blends are formulated by combining biodiesel with diesel in various ratios to power diesel engines, effectively reducing the emission of harmful pollutants into the environment [43]. Typically, conventional feedstocks used for blending with diesel include food crops and plants. However, utilizing food crops as feedstocks for biodiesel blends can raise concerns regarding food scarcity and competition [44]. Similarly, relying on plant-based feedstocks like palm oil can contribute to ongoing deforestation, which in turn intensifies the greenhouse effect [45]. Therefore, the biodiesel derived from lipid extraction utilizing a two-step process, which involves a 5-minute ultrasound-assisted DES pretreatment of microalgal biomass followed by ultrasound-assisted solvent extraction, presents an excellent option for governmental bodies to consider for commercialization as a novel feedstock for biodiesel blending purposes.

### 3.2 Value-Added green biorefinery product II: Hydroponic fertilizer production

**Fig. 2:**
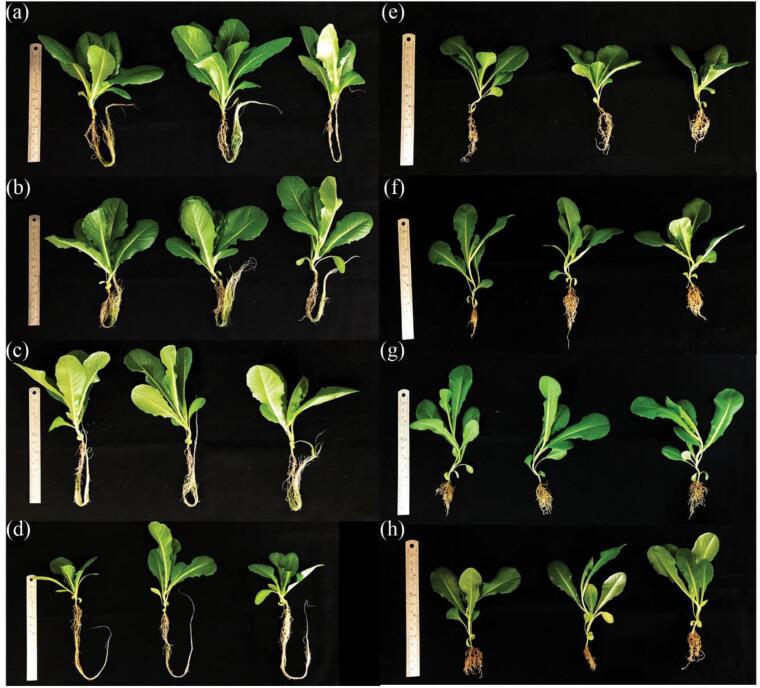
Growth of lettuce after 28 days of cultivation in deep water culture system under commercial nutrient solution AB alone (a-d) and nutrient solution AB mixed with microalgal aqueous extract (AE) (e-h) with different electrical conductivity levels in 1800 µS/cm (a and e), 900 µS/cm (b and f), 450 µS/cm (c and g), 225 µS/cm (d and h).

**Fig. 3:**
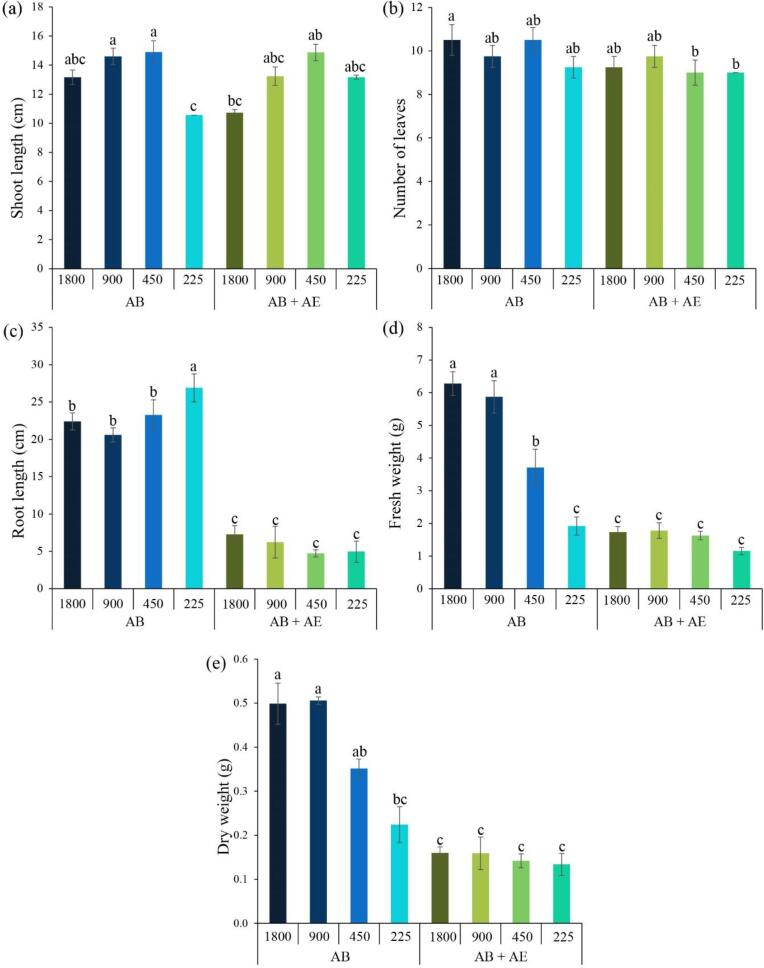
Shoot length (a), number of leaves (b), root length (c), fresh weight (d) and dry weight (e) of lettuce after 28 days of cultivation in deep water culture system under commercial nutrient solution AB alone (AB) and nutrient solution AB mixed with microalgal aqueous extract (AE) (AB + AE) with different electrical conductivity levels (225–1800 µS/cm).

Following the optimized lipid extraction through a two-step procedure consisting of a 5-minute ultrasound-assisted DES pretreatment of microalgal biomass followed by ultrasound-assisted solvent extraction, the lipid-extracted microalgal biomass residues (referred to as LMBRs) were subjected to a water extraction method to recover the aqueous extract (AE). This specific extraction approach resulted in the recovery of approximately 25.23 % (equivalent to 0.50 g of AE per liter of working volume) from the LMBRs. In the cultivation of actinobacteria-inoculated lettuce under a laboratory hydroponic deep water culture system, the nutrient solution AE was incorporated in combination with the commercially available hydroponic fertilizer AB. This was done to achieve a desired electrical conductivity (EC) range of 225–1800 µS/cm. Control experiments were also carried out using nutrient solution AB alone, maintaining the same electrical conductivity range. The results demonstrated that lettuce grown exclusively in nutrient solution AB exhibited a greater shoot length range of 10.75–14.33 cm compared to lettuce cultivated in the combined nutrient solution of AB + AE, which exhibited a slightly lower shoot length range of 10.58–13.63 cm (Fig. 2, Fig. 3a), while the quantity of lettuce leaves in the nutrient solution AB alone ranged from 9.25 to 10.50 leaves, showing a slight numerical superiority over the number of lettuce leaves observed in the combined nutrient solution of AB + AE, which ranged from 9.00 to 9.75 leaves (Fig. 2, Fig. 3b). Lettuce cultivated exclusively in the commercial nutrient solution AB likely experienced favorable shoot development and leaf production due to the specific combination and concentration of nutrients provided by the designed formulation. This balanced nutrient composition in the commercial hydroponic fertilizer is designed to optimize shoot length and leaf number by supplying essential nutrients and growth-promoting substances [46]. On the other hand, the addition of microalgal AE to the commercial nutrient solution AB may have caused changes in the nutrient composition or balance within the solution. These alterations in nutrient availability and ratios could have influenced the growth of the lettuce plants [47], resulting in a slightly lower shoot length and number of leaves compared to those grown exclusively in the commercial hydroponic fertilizer. When employing the nutrient solution AB + AE, the shoot length of lettuce cultivated in the nutrient solution with EC ranging from 225 to 900 µS/cm exhibited an increase of 1.12–1.29 times in comparison to the EC of 1800 µS/cm (Fig. 3a). It is possible that high EC levels can result in osmotic stress on plants, limiting their growth and development [48]. By reducing the EC values, the osmotic stress on the lettuce plants is alleviated, allowing for more optimal growth conditions [49]. This reduction in stress can contribute to the observed increase in shoot length. However, a slight increase in the number of lettuce leaves was observed when the EC value was reduced to 900 µS/cm. A further decrease in EC to 225 µS/cm did not yield any noticeable improvement in the number of lettuce leaves (Fig. 3b). It is possible that the nutrient availability at the high EC was sufficient to support slight increases in leaf production, while reducing the EC further may not have provided any additional benefits in terms of nutrient uptake or leaf development [50].

In terms of root length, the maximum value was observed in lettuce cultivated in the nutrient solution AB + AE with EC of 1800 µS/cm, reaching 7.30 cm. However, as the EC values were reduced to the range of 225–900 µS/cm, a subsequent decrease in root length was observed, with reductions of 1.17–1.54 times (4.73–7.30 cm). An explanation for the longer root length at higher EC values is that high EC values impose osmotic pressure on plant roots. This pressure differential encourages water uptake by the roots, promoting cell expansion and elongation [51]. Some plant species, including lettuce, exhibit a response known as “osmotic adjustment” when exposed to high EC levels [52]. As a result, the roots may exhibit greater growth and longer lengths. However, when lettuce was cultivated in the nutrient solution AB, a substantial increase in root length (20.60–26.90 cm) was observed compared to lettuce grown solely in the nutrient solution AB + AE. This enhancement in root length was significant, with fold increases ranging from 3.07 to 5.41 (Fig. 3c). This might be due to the significance of balanced nutrient compositions in promoting plant growth and development [46], [47]. One possible explanation for this observation is that elevated pH levels, known as alkaline pH, can restrict the growth of hydroponic lettuce roots, as indicated in Table 6. In alkaline conditions, certain nutrients, such as iron, manganese, and zinc, tend to become less soluble and less accessible to the roots [53]. It can alter the activity of specific transporters responsible for nutrient absorption, reducing the efficiency of nutrient uptake [54]. This nutrient deficiency can hinder root growth and development, subsequently limiting overall root length.

**Table 6:** Change in pH during lettuce cultivation in nutrient solution AB alone and nutrient solution AB + AE under difference electrical conductivity (EC) levels (225–1800 µS/cm).

Days	Nutrient solution AB alone				Nutrient solution AB + AE			
	1800	900	450	225	1800	900	450	225
0	6.18 ± 0.00	6.47 ± 0.00	6.62 ± 0.00	6.94 ± 0.00	5.86 ± 0.00	5.98 ± 0.00	6.04 ± 0.00	6.12 ± 0.00
7	6.67 ± 0.07	6.66 ± 0.22	7.17 ± 0.05	7.34 ± 0.01	8.20 ± 0.10	8.50 ± 0.08	8.48 ± 0.08	8.35 ± 0.08
14	6.75 ± 0.07	7.15 ± 0.13	7.59 ± 0.04	7.63 ± 0.06	8.63 ± 0.04	8.79 ± 0.04	8.62 ± 0.03	8.61 ± 0.09
21	6.55 ± 0.25	7.37 ± 0.23	7.97 ± 0.23	7.65 ± 0.14	8.60 ± 0.02	8.72 ± 0.06	8.52 ± 0.03	8.51 ± 0.04
28	7.10 ± 0.82	8.16 ± 0.38	8.10 ± 0.50	7.39 ± 0.05	8.26 ± 0.05	8.54 ± 0.03	8.31 ± 0.02	8.27 ± 0.07

When examining the fresh and dry weights of shoot and root, an evident correlation was observed between the trends displayed in the fresh weights (Fig. 3d) and the corresponding trends observed in the dry weights (Fig. 3e) of lettuce. The fresh weight (ranging from 1.92 to 6.28 g) and dry weight (ranging from 0.22 to 0.50 g) of shoot and root of lettuce cultivated in nutrient solution AB were found to be greater compared to lettuce grown in nutrient solution AB + AW. In the case of fresh weight, lettuce grown in nutrient solution AB + AW exhibited a range of 1.16 to 1.78 g, while for dry weight, the range was 0.13 to 0.16 g. As we mentioned above, the balanced nutrient composition in commercial hydroponic fertilizers ensures that all essential nutrients are provided in appropriate proportions. This balance of nutrients supports healthy and vigorous plant growth, leading to increased biomass accumulation in both the shoot and root [46]. On the other hand, the addition of microalgal aqueous extract might have altered the nutrient balance, potentially impacting nutrient availability and uptake, resulting in lower fresh and dry weights compared to plants grown exclusively in the commercial hydroponic fertilizer. Commercial hydroponic fertilizers often contain growth-promoting substances, such as plant hormones and organic compounds, that stimulate plant growth and development. These substances can enhance cell division, elongation, and overall biomass accumulation [55]. In contrast, the microalgal aqueous extract may not contain the same growth-promoting substances, leading to a reduced growth response in lettuce, reflected in lower fresh and dry weights.

In addition, the fresh weight of the shoot and root of lettuce cultivated in nutrient solutions AB and AB + AE exhibited a notable decrease. Specifically, the fresh weight of lettuce grown in nutrient solution AB decreased from 6.28 g to 1.92 g, while the fresh weight of lettuce grown in nutrient solution AB + AE decreased from 1.73 g to 1.16 g (Fig. 3d). These reductions were observed as EC values were systematically reduced from 1800 µS/cm to 225 µS/cm. Likewise, a notable decrease was observed in the dry weight of both the shoot and root of lettuce when the EC values were reduced. In particular, lettuce cultivated in nutrient solution AB exhibited a decrease in dry weight from 0.50 g to 0.22 g, while lettuce grown in nutrient solution AB + AE displayed a decrease from 0.16 g to 0.13 g (Fig. 3e). Lower EC values indicate a lower concentration of salts, including essential nutrients. As a result, the nutrient availability in the solution decreases, potentially leading to nutrient deficiencies [50]. Insufficient nutrient supply can restrict plant growth, resulting in lower fresh and dry weights of the shoot and root. Lower EC values also diminish the osmotic gradient, which can impede the efficient uptake of nutrients by the plant roots [48]. Osmotic stress can hinder water uptake by the roots and disrupt cell functioning [48], [50], leading to reduced growth and lower fresh and dry weights of both the shoot and root. Also, lower EC values can throw off the balance of hormones that control growth, like auxins and cytokinins, which are very important for root and shoot growth [56]. This hormonal imbalance can contribute to reduced growth and biomass accumulation.

**Fig. 4:**
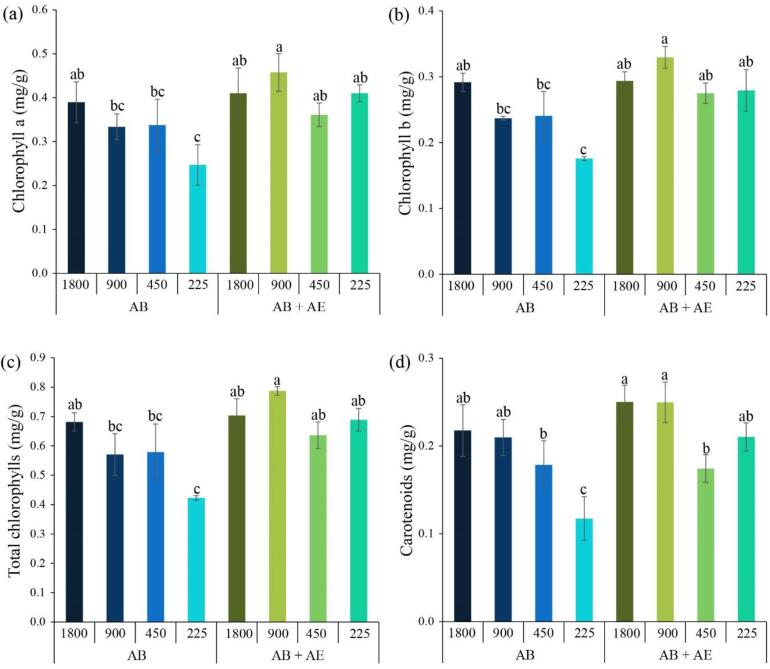
Chlorophyll *a* (a), chlorophyll *b* (b), total chlorophylls (c), and carotenoids (d) of lettuce after 28 days of cultivation in deep water culture system under commercial nutrient solution AB alone (AB) and nutrient solution AB mixed with microalgal aqueous extract (AE) (AB + AE) with different electrical conductivity levels (225–1800 µS/cm).

Based on the levels of photosynthetic pigments observed in Fig. 4, a similar trend was observed in the concentrations of chlorophyll *a*, chlorophyll *b*, total chlorophylls, and carotenoids in lettuce. Specifically, the levels of these photosynthetic pigments in lettuce cultivated in nutrient solutions AB + AE (0.36–0.46 mg/g for chlorophyll *a*, 0.28–0.33 mg/g for chlorophyll *b*, 0.64–0.79 mg/g for total chlorophylls, and 0.18–0.25 mg/g for carotenoids) were higher compared to lettuce cultivated in nutrient solutions AB (0.25–0.39 mg/g for chlorophyll *a*, 0.18–0.29 mg/g for chlorophyll *b*, 0.42–0.68 mg/g for total chlorophylls, and 0.12–0.22 mg/g for carotenoids). Generally, the enhanced quantity of photosynthetic pigments can contribute to the efficient capture and utilization of light energy by reflecting absorbed light, transmitting it, dispersing it, and spreading it through the leaves. This has immediate implications for the rate of photosynthesis and plant growth, ultimately influencing yield and the utilization efficiency of light [57]. The elevated levels of photosynthetic pigments observed in this study may indicate a potential adaptive response of lettuce to address nutritional limitations, especially when faced with low-nutrient stress or imbalanced nutrient conditions [58]. This adaptive strategy may involve the expansion of the light-harvesting complex, allowing lettuce to enhance its capacity for light absorption. As a result, this adaptive response can lead to an increased synthesis of pigments, ultimately contributing to improved photosynthetic efficiency and plant performance under challenging nutrient conditions.

As depicted in Fig. 4, a decrease in EC levels can indeed restrict the synthesis of photosynthetic pigments in lettuce. This can be attributed to compromised nutrient availability when EC levels decrease, resulting in limited uptake and reduced availability of essential elements. The synthesis of photosynthetic pigments, including chlorophylls (such as chlorophyll *a* and chlorophyll *b*) and carotenoids, relies heavily on the presence of key nutrients like nitrogen, phosphorus, magnesium, and iron [59]. When nutrient availability is constrained, plants must prioritize the allocation of limited resources to sustain growth and development. In this scenario, maintaining a higher chlorophyll content becomes crucial for plants to facilitate the process of photosynthesis, which is vital for their survival [60]. Consequently, the synthesis of carotenoids may be comparatively limited under low nutrient conditions. The limited availability of nutrients and the subsequent impact on photosynthetic pigment synthesis can explain the observed relationship between decreased EC levels and diminished production of photosynthetic pigments in lettuce. These findings emphasize the importance of maintaining appropriate nutrient levels and balance to support optimal pigment synthesis and, ultimately, efficient photosynthesis and plant growth.

While the utilization of AE in nutrient solution AB + AE demonstrated a relatively lesser promotion of lettuce growth in comparison to nutrient solution AB alone, it is noteworthy that the AE derived from LMBRs holds potential as a component of hydroponic fertilizer. This is due to the ability of lettuce to thrive and be cultivated in the presence of AE. Further research and experimentation are necessary to determine the optimal dosage and application strategies of AE to maximize its benefits in hydroponic lettuce cultivation. Furthermore, it is worth noting that while AE may find applicability in laboratory hydroponic experiments, its utility is somewhat constrained by environmental factors. Consequently, it is advisable to consider the exploration of greenhouse and outdoor experimental settings in future investigations to enhance practicality and real-world relevance.

## 4 Conclusion

This study demonstrates successful production of biorefinery co-products using ultrasonically assisted deep eutectic solvent (DES)-pretreated microalgal *Chlorella* biomass. A two-step procedure involving 5-minute ultrasound-assisted DES pretreatment and solvent extraction effectively extracts lipids from *Chlorella* biomass, yielding biodiesel-quality lipids by 2.10-folds compared to a one-step process that combined ultrasound-assisted DES pretreatment and solvent extraction. Moreover, the aqueous extract recovered from lipid-extracted microalgal biomass residues (LMBRs) shows potential as a component in hydroponic biofertilizer production, supporting lettuce growth. Therefore, microalgal biorefinery holds immense potential in improving the economic viability and environmental sustainability of interconnected sectors, including renewable energy and agriculture.

## CRediT authorship contribution statement

**Antira Wichaphian:** Methodology, Validation, Formal analysis, Investigation, Data curation, Writing – original draft, Writing – review & editing, Visualization, Project administration. **Nanthakrit Sriket:** Formal analysis, Investigation, Writing – review & editing. **Sritip Sensupa:** Writing – review & editing. **Jeeraporn Pekkoh:** Writing – review & editing. **Wasu Pathom-aree:** Writing – review & editing, Funding acquisition. **Yupa Chromkaew:** Writing – review & editing, Supervision. **Nakarin Suwannarach:** Writing – review & editing. **Jaturong Kumla:** Writing – review & editing. **Benjamas Cheirsilp:** Writing – review & editing, Supervision. **Sirasit Srinuanpan:** Conceptualization, Methodology, Software, Resources, Data curation, Writing – original draft, Writing – review & editing, Visualization, Supervision, Funding acquisition.

## Declaration of Competing Interest

The authors declare that they have no known competing financial interests or personal relationships that could have appeared to influence the work reported in this paper.
